# The interaction of fatigue cracks with a residual stress field using thermoelastic stress analysis and synchrotron X-ray diffraction experiments

**DOI:** 10.1098/rsos.171100

**Published:** 2017-11-08

**Authors:** Khurram Amjad, David Asquith, Eann A. Patterson, Christopher M. Sebastian, Wei-Chung Wang

**Affiliations:** 1 School of Engineering, University of Liverpool, Liverpool L69 3GH, UK; 2 Department of Power Mechanical Engineering, National Tsing Hua University, Taiwan, ROC; 3 Materials and Engineering Research Institute, Sheffield Hallam University, Sheffield S1 1WB, UK

**Keywords:** cold expansion, fastener holes, residual stresses, thermoelastic stress analysis, synchrotron X-ray diffraction, fatigue cracks

## Abstract

This article presents an experimental study on the fatigue behaviour of cracks emanating from cold-expanded holes utilizing thermoelastic stress analysis (TSA) and synchrotron X-ray diffraction (SXRD) techniques with the aim of resolving the long-standing ambiguity in the literature regarding potential relaxation, or modification, of beneficial compressive residual stresses as a result of fatigue crack propagation. The crack growth rates are found to be substantially lower as the crack tip moved through the residual stress zone induced by cold expansion. The TSA results demonstrated that the crack tip plastic zones were reduced in size by the presence of the residual compressive stresses induced by cold expansion. The crack tip plastic zones were found to be insignificant in size in comparison to the residual stress zone resulting from cold expansion, which implied that they were unlikely to have had a notable impact on the surrounding residual stresses induced by cold expansion. The residual stress distributions measured along the direction of crack growth, using SXRD, showed no signs of any significant stress relaxation or redistribution, which validates the conclusions drawn from the TSA data. Fractographic analysis qualitatively confirmed the influence on crack initiation of the residual stresses induced by the cold expansion. It was found that the application of single compressive overload caused a relaxation, or reduction in the residual stresses, which has wider implications for improving the fatigue life.

## 1. Introduction

The manufacturing of aerospace structures requires thousands of fastener holes to be drilled for the purpose of assembly. Fatigue cracks can initiate from such holes, thereby reducing the overall integrity of aerospace structures. The split sleeve cold expansion of holes is one of the most widely used cold-working processes in the aerospace industry and is employed to improve the fatigue performance of new and old airframes. In this process, a hardened steel mandrel, with an oversized head, is passed through an initially undersized hole to cause plastic expansion. As the mandrel head is removed after the expansion, a ring of compressive residual stresses is developed as a result of the spring-back effect from the surrounding elastic material. An internally lubricated split sleeve resides on the mandrel shank to allow the cold expansion to be carried out with access to only one side of the component. It also ensures that there is no direct contact of the mandrel head with the internal hole edge, which minimizes the out-of-plane distortion during cold expansion. The specimen faces from which the mandrel enters and exits the specimen during cold expansion are referred to as the mandrel entry and exit faces, respectively.

Life predictions for fatigue cracks emanating from cold-expanded holes involve the determination of the effective stress intensity factor range (Δ*K*_eff_) by superimposing stress intensity factor due to the residual stress field resulting from cold expansion on the corresponding one due to the applied mechanical loads, typically using the weight function method [1–7], and the subsequent use of the evaluated Δ*K*_eff_ values in appropriate theoretical relations, similar to Paris' Law, which relates Δ*K*_eff_ to the crack growth rate. This approach intrinsically assumes that the initial residual stress field developed by cold expansion does not change due to propagation of a fatigue crack. Various researchers [1,7–9] have highlighted that the residual stresses could potentially redistribute as the fatigue crack grows and this should be taken into account for reliable fatigue life predictions. Therefore, it is important to develop a clear understanding about the potential for and causes of any redistribution of these residual stresses. Various aspects related to the fatigue performance of cold-expanded holes have been studied extensively over the past three and a half decades; however, few investigations [10–18] have focused on the potential redistribution or relaxation of beneficial compressive residual stresses resulting from either fatigue loading or due to the propagation of fatigue cracks from such holes. Cannon *et al*. [10] were the first to state that the application of large compressive loads can cause the redistribution or relaxation of these compressive residual stresses. Stefanescu *et al*. [11] later reaffirmed their conclusions by determining the residual stress relaxation close to the hole edge, resulting from compressive overloads, using a laboratory X-ray diffraction technique. It is, therefore, well understood that the fatigue loads, causing large-scale plastic deformation at the hole edge, could significantly alter the initial residual stress distribution. Compressive loads are particularly detrimental in this context, because only a small magnitude load is required to cause the highly compressive residual stresses at the hole edge to exceed the yield stress.

Some researchers [12–16] have reported a reduction in the residual stresses under fatigue loads which were not expected to cause large-scale plastic deformation at the hole edge. Herman & Moffat [12] used a destructive Sachs method to determine the relaxation at such fatigue loads in aluminium–lithium alloy specimens; though, no attempt was made to explain the cause of this decay. Özdemir & Edwards [13] used a similar Sachs technique to show pronounced stress relaxation in 7075 aluminium specimens. The cause of this relaxation was reported to be the growth of short fatigue cracks from the hole edge, which were ultimately arrested as the residual stress distribution stabilized. Their results, therefore, indicate that the plastic zone associated with the crack tip could potentially affect the initial distribution of residual stresses. Stefanescu *et al*. [14,15] investigated the effect of a fatigue crack on the initial residual stress distribution in specimens, similar to those used by Özdemir & Edwards [13], using both laboratory and synchrotron X-ray diffraction techniques. In their work, a fatigue crack was grown from a 0.2 mm through-thickness electric discharge machined (EDM) notch at the edge of the hole. A relatively less-pronounced relaxation of residual stresses close to the hole edge was reported. This could be attributed to the presence of the notch because it both involves material removal with some associated plastic work and a geometric discontinuity in the hole circumference that is significant enough to cause interruption of the load paths, and thus, redistribution of the residual stresses around its vicinity.

Lacarac *et al*. [18] used a different approach to investigate the issue of residual stress relaxation by measuring the crack opening stress, *σ*_op_ at different crack lengths. The values for *σ*_op_ were found to be constant for all crack lengths. In a more recent investigation by Backman *et al*. [19], crack opening displacements along crack flanks were found to be constant across the compressive residual stress zone, which is in agreement with the results reported by Lacarac *et al*. [18]. The findings of these investigations [18,19], therefore, imply that the residual stress distribution does not necessarily relax as a result of fatigue crack propagation, which is contrary to the conclusions drawn by Özdemir & Edwards [13] and Stefanescu *et al*. [14,15]. The experimental study presented in this article investigates the propagation of fatigue cracks emanating from cold-expanded holes and their influence on the surrounding residual stresses, with the aim of resolving the above-mentioned differences found in the literature by establishing the reasons for the potential redistribution of these residual stresses.

## 2. Background

Two experimental techniques were used in this work, i.e. thermoelastic stress analysis (TSA) and synchrotron X-ray diffraction (SXRD). TSA was employed to study the behaviour of fatigue cracks during propagation; whereas SXRD was used to measure residual stresses around un-cracked and cracked cold-expanded holes to determine whether any significant redistribution of these residual stresses occurred. Fractographic analysis was also performed to qualitatively analyse the influence of residual stresses on fatigue crack initiation.

### 2.1. Thermoelastic stress analysis technique

TSA is a non-contact technique used to determine the stresses from the surface of a cyclically loaded specimen by measuring the thermoelastic effect. The theoretical basis for the thermoelastic effect was first proposed by Lord Kelvin in 1853 by relating the temperature change to the elastic deformation. The detailed mathematical theory underpinning TSA can be found in a review by Pitarresi & Patterson [20]. The generalized form of the relation that relates the change in temperature of an elastic solid to its change in strain is as follows:

2.1 $$\Delta T=\frac{T}{\rho C_{\varepsilon }}\sum \frac{\partial \sigma_{ij}}{\partial T}\Delta \varepsilon_{ij}+\frac{Q}{\rho C_{\varepsilon }},$$

where Δ*T* is the temperature change, *T* is the absolute temperature, *ρ* is the density, *C*_ε_ is the specific heat capacity at constant strain, *σ*_ij_ and *ε_ij_* are the stress and stain tensors, respectively, and *Q* is the heat input. TSA is usually performed by loading the specimen cyclically, at a suitably high frequency to ensure adiabatic conditions, which allows the second term in equation (2.1) to be ignored. For an isotropic material in a state of plane stress and assuming adiabatic, reversible conditions, equation (2.1) can be simplified to:

2.2 $$\Delta T=-\frac{\alpha T}{\rho C_{p}}\Delta (\sigma_{11}+\sigma_{22}),$$

where *α* is the coefficient of linear thermal expansion and *C*_p_ is the specific heat capacity at constant pressure. The variation in temperature on the surface of a cyclically loaded specimen can be measured by an infrared detector in terms of the voltage output, *S* of the detector. Therefore, the working form of the relationship used for practical TSA is as follows:

2.3 $$AS=\Delta (\sigma_{11}+\sigma_{22}),$$

where *S* is the output signal from the infrared detector, which corresponds to the thermoelastic effect, and *A* is the calibration constant, which is a function of both the material properties and the detector parameters. The calibration constant, *A* is usually determined experimentally by obtaining the detector signal from a region on the specimen's surface with a known stress state. In order to relate the temperature changes to the voltage variations from an infrared detector, the signal from a detector needs to be correlated with a reference signal representing the loading frequency of the specimen, which is obtained from the fatigue test machine. The output data from a TSA set-up, therefore, is in the form of a vector, whose magnitude represents the thermoelastic response that is proportional to the temperature change and whose orientation represents the phase shift between the thermoelastic response and the reference signal. Under linear and adiabatic conditions, the phase of the thermoelastic response is uniform over a specimen's surface. The stress intensity factor range (Δ*K*) at the tip of fatigue cracks can be evaluated experimentally from the thermoelastic data using a methodology developed by Tomlinson *et al*. [21], which was further improved and implemented in the software algorithm, FATCAT by Diaz *et al.* [22]. In this methodology, a mathematical model describing the distribution of the sum of principal stresses around a crack tip, which is based on Muskhelishvili's approach [23], is fitted to the thermoelastic data collected from the singularity-dominated elastic zone around the crack tip. The location of the crack tip and the extent of the singularity-dominated region can be determined using the methodology described by Diaz *et al*. [22].

### 2.2. Synchrotron X-ray diffraction technique

Modern synchrotron radiation sources are capable of providing a high intensity, coherent, monochromatic, X-ray beam with a small spot size, and have great potential for engineers to make non-destructive, high resolution, residual stress measurements in metallic components at much greater penetrations depths [24] than was previously possible. The conventional approach for measuring residual stresses using SXRD, referred to as the *θ*/2*θ* scanning method [24], was used in this experimental study. This technique utilizes the phenomenon of shifts in Bragg's diffracted intensity peak to determine the residual strains and is briefly explained here. The X-rays are diffracted from the atomic planes of a crystal lattice based on the well-known Bragg's law:

2.4 nλ=2dsin⁡θ,

where *λ* is the wavelength, *n* is the order number of the wavelength, *d* is the distance between atomic planes and *θ* is the diffraction angle. The angle at which a diffracted intensity peak is detected is measured using a diffractometer and the lattice spacing is thus calculated from Bragg's Law. The presence of residual elastic stresses causes a change in the lattice spacing and this causes a change in the angle at which the diffracted intensity peak is detected. Therefore, for the purpose of evaluating the residual strains (*ε*), the strain-free lattice spacing (*d*_0_) needs to be determined as well. The residual strain can be calculated from the change in lattice spacing using the following equation:

2.5 $$\varepsilon =\frac{d-d_{0}}{d_{0}},$$

The suitability of this approach for measuring residual stresses around cold-expanded holes has been previously investigated by Stefanescu *et al*. [15]. The measurements from SXRD using this method were compared with neutron diffraction and laboratory X-ray diffraction results and were found to be in good agreement [15].

## 3. Experimental work

Experiments were conducted using aluminium specimens (6.02 mm thickness) with central holes (diameter 6.36 mm). In some of these specimens the hole was cold-worked to expand it by 3.5% using a mandrel in a split sleeve, while other specimens were left as control samples with un-expanded holes. Digital image correlation was used during the cold expansion to measure the induced strains; subsequently fatigue loading was applied to the specimens and both the stress field and extent of the plasticity monitored using thermoelastic stress analysis on one face while a pair of digital cameras was used to monitor fatigue cracks on the other face. A small subset of both the control and cold-expanded specimens were examined using synchrotron X-ray diffraction to evaluate the residual strain fields associated with both the cold expansion process and fatigue crack propagation. Finally, optical and scanning electron microscopes were used to examine the fracture surfaces in a small number of specimens from the control set and cold-expanded set. A detailed description of these experiments is provided below while table 2 provides an overview of the fatigue loading applied to the specimens.

### 3.1. Cold expansion and fatigue test procedures

**Figure 1.**
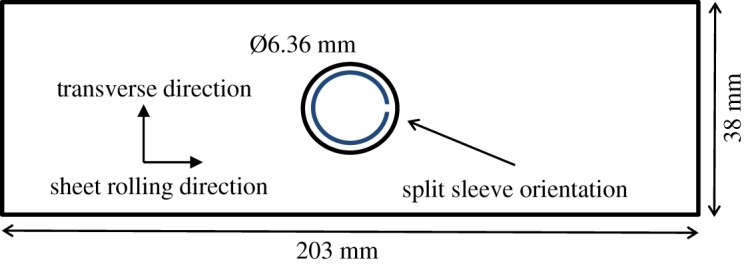
Schematic of centrally drilled hole specimen with the split sleeve shown in the hole (not to scale).

A total of 23 specimens were machined, with the geometry shown in figure 1, from a 2024-T351 aluminium plate with a nominal thickness of 0.25′′ (6.35 mm). The thickness of the fabricated specimens was measured to be 6.02 mm. The mechanical properties of the aluminium plate, provided in table 1, were determined by performing tensile tests conforming to ASTM standards [25,26]. A central hole of 6.36 mm diameter was drilled in all the specimens giving a thickness-to-diameter ratio of 0.95. Cold expansion was performed in 17 of the specimens using a split sleeve cold expansion kit from Fatigue Technology Inc., USA [27], while the remaining six specimens were left with un-expanded holes. During cold expansion, the split in the sleeve was positioned along the longitudinal direction of the specimen as shown in figure 1. The combination of the oversized mandrel head and split sleeve thickness provided a maximum interference of 4.6%. The diameter of the expanded hole after mandrel removal was measured to be 6.58 mm, providing a retained expansion of 3.5%.

**Table 1.** Mechanical properties of the 2024-T351 aluminium plate.

	0.2% proof stress (MPa)	tensile strength (MPa)	elastic modulus (GPa)	elongation (%)	Ramberg–Osgood strain hardening parameter, *n*
rolling direction	315	505	72	19.3	7.0
transverse direction	300	485	71	20.7	6.20

**Figure 2.**
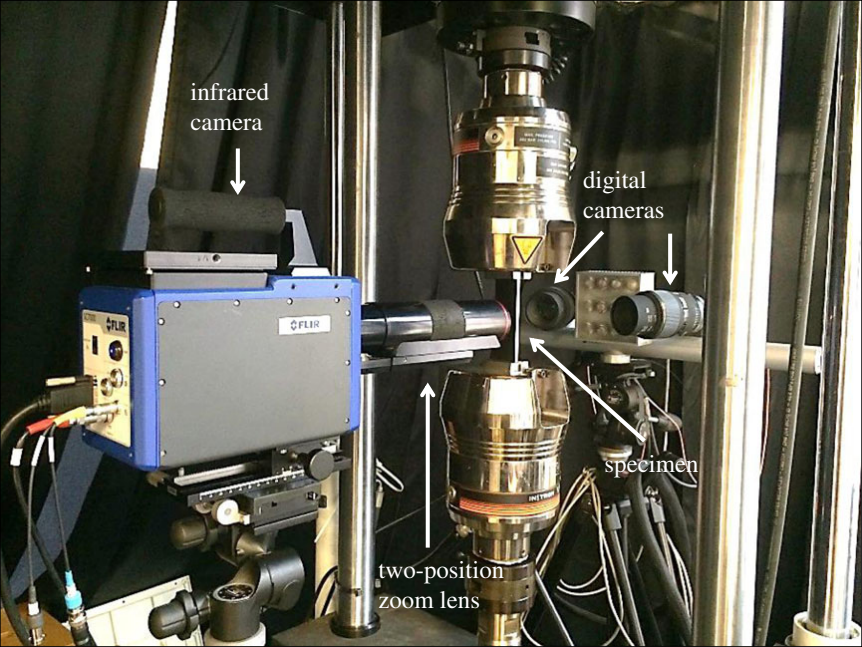
Experiment set-up for the fatigue tests.

Fatigue tests were performed in an Instron servo-hydraulic test machine equipped with a 100 kN load cell. The experimental set-up used for the fatigue tests is shown in figure 2. On the mandrel entry side of the cold-expanded specimens, an infrared camera (FLIR 7650; FLIR Systems Inc., USA), with a InSb detector array of 640 × 512 pixels, was employed to simultaneously monitor the cracks and capture the thermoelastic response. The camera is capable of acquiring images using the full sensor at a maximum frame rate of 100 Hz. For this experiment, the camera was operated at a frame rate of 250 Hz with a reduced active sensor window of 320 × 256 pixels. A two-position zoom lens (Stressphotonics Ltd, USA) was used and provided a spatial resolution of 29.6 µm pixel^−1^. On the mandrel exit side, the cracks were monitored optically using a pair of digital cameras (Guppy PRO F-125; Allied Vision Technologies, Germany) with a resolution of 1292 × 964 pixels. The digital cameras were mounted with a pair of identical Sigma macro lenses of 105 mm focal length, providing a spatial resolution of 8 µm pixel^−1^. For the un-expanded specimens, the specimen faces facing the infrared camera and the pair of digital cameras will be referred to as the front and back faces, respectively, in the later sections.

Two different levels of maximum applied load were used for fatigue loading of the cold-expanded and the un-expanded specimens. For the cold-expanded specimens, the maximum applied load of 38.9 kN was used compared with 34.3 kN for the un-expanded ones. These load levels corresponded to the maximum remote nominal stresses of 170 and 150 MPa, respectively. The primary reason for using a lower maximum applied load for the un-expanded specimens was to reduce the crack growth rate in order to make it possible to manually track the tip of a growing crack and simultaneously capture the thermoelastic response from the crack tip region using an infrared camera; while a higher load was needed for the cold-expanded specimens to allow the tests to be conducted in a practical time period. All fatigue tests were performed using a load ratio of 0.1 and a frequency of 19 Hz. The fatigue loads applied to the cold-expanded specimens were chosen to ensure that the maximum hoop stress at the hole edge does not exceed the yield stress in order to avoid large-scale plastic deformation. The maximum hoop stress at the hole edge, *σ*_h,max_ was calculated by linear superposition of the hoop stress resulting from the maximum applied load (*σ*_h,app_) and the compressive residual hoop stress developed from cold expansion (*σ*_h,res_). *σ*_h,app_ was evaluated by multiplying the stress concentration factor of 3.1 at the hole edge, determined for the specimen geometry shown in figure 1 using an empirical relation provided in Peterson's SCF handbook [28], with the maximum remote stress (*σ*_R,max_). *σ*_h,res_ was taken as the residual hoop stress value obtained from the SXRD measurements close to the hole edge. It is pertinent to mention here that *σ*_h,max_ is a theoretical value for superimposed elastic stresses, which does not necessarily represent the actual magnitude of hoop stresses being experienced at the hole edge; and its sole purpose is to determine whether the applied loads are expected to cause any yielding.

All six un-expanded specimens were loaded in fatigue until failure using the above-mentioned fatigue loading parameters. Out of the 17 cold-expanded specimens, a set of six specimens were loaded in fatigue until failure. For another set of three cold-expanded specimens, fatigue loading was stopped prior to failure for the purpose of performing residual stresses measurements on them. In two of the cold-expanded specimens, a single load cycle with maximum compressive loads of −21.2 and −28.6 kN was applied, corresponding to the maximum remote nominal stresses of −92.7 and −125 MPa, respectively. These compressive loads were chosen to represent the service loads on the wing pivot fitting of a fighter aircraft as reported by Pell *et al*. [29]. After examining these specimens in a synchrotron, they were also loaded in fatigue until failure. No monotonic or fatigue loads were applied on the remaining set of six cold-expanded specimens. They were examined in a synchrotron to determine the uncertainty in the measurements of residual stress. The details of the fatigue test programme described above are summarized in table 2.

**Table 2.** Summary of fatigue tests performed in the experimental study. *σ*_h,max_ is the maximum hoop stress at the hole edge which was determined by linear superposition of the hoop stress resulting from the applied load (*σ*_h,app_) and the compressive residual stress developed from cold expansion (*σ*_h,res_).

specimen ID	hole expansion	applied loads	max. hoop stress at hole edge (σ_h,max_)	remarks
U1–U6	un-expanded	fatigue parameters:	+465	— fatigue loaded until failure
		— *σ*_R,max_ = 150 MPa — *R* = 0.1 — Fr = 19 Hz		
C1–C6	cold-expanded	fatigue parameters:	+185	— fatigue loaded until failure
		— *σ*_R,max_ = 170 MPa — *R* = 0.1 — Fr = 19 Hz		
C7^a^	cold-expanded	fatigue parameters:	+185	— loading stopped after 50 k cycles — no cracks observed
		— *σ*_R,max_ = 170 MPa — R = 0.1 — Fr = 19 Hz		
C8^a^	cold-expanded	fatigue parameters:	+185	— loading stopped after 150 k cycles — left and right hole edge cracks of 2.1 and 2.4 mm measured on the specimen mandrel entry face
		— *σ*_R,max_ = 170 MPa — *R* = 0.1 — Fr = 19 Hz		
C9^a^	cold-expanded	fatigue parameters:	+185	— loading stopped after 400 k cycles — left and right hole edge cracks of 3.8 and 3 mm measured on the specimen mandrel entry face
		— *σ*_R,max_ = 170 MPa — *R* = 0.1 — Fr = 19 Hz		
C10^a^	cold-expanded	initial single compressive load:	−627	— fatigue loaded until failure after performing SXRD measurements
		— *σ*_c _ = −92.7 MPa		
		fatigue parameters:		
		— *σ*_R,max_ = 170 MPa — *R* = 0.1 — Fr = 19 Hz		
C11^a^	cold-expanded	initial single compressive load:	−730	— fatigue loaded until failure after performing SXRD measurements
		— *σ*_c_ = −125 MPa		
		Fatigue parameters:		
		— *σ*_R,max_ = 170 MPa — *R* = 0.1 — Fr = 19 Hz		
C12–C17^a^	cold-expanded	—	—	— no loads applied

^a^Examined in synchrotron.

### 3.2. Synchrotron X-ray diffraction experiment procedures

**Figure 3.**
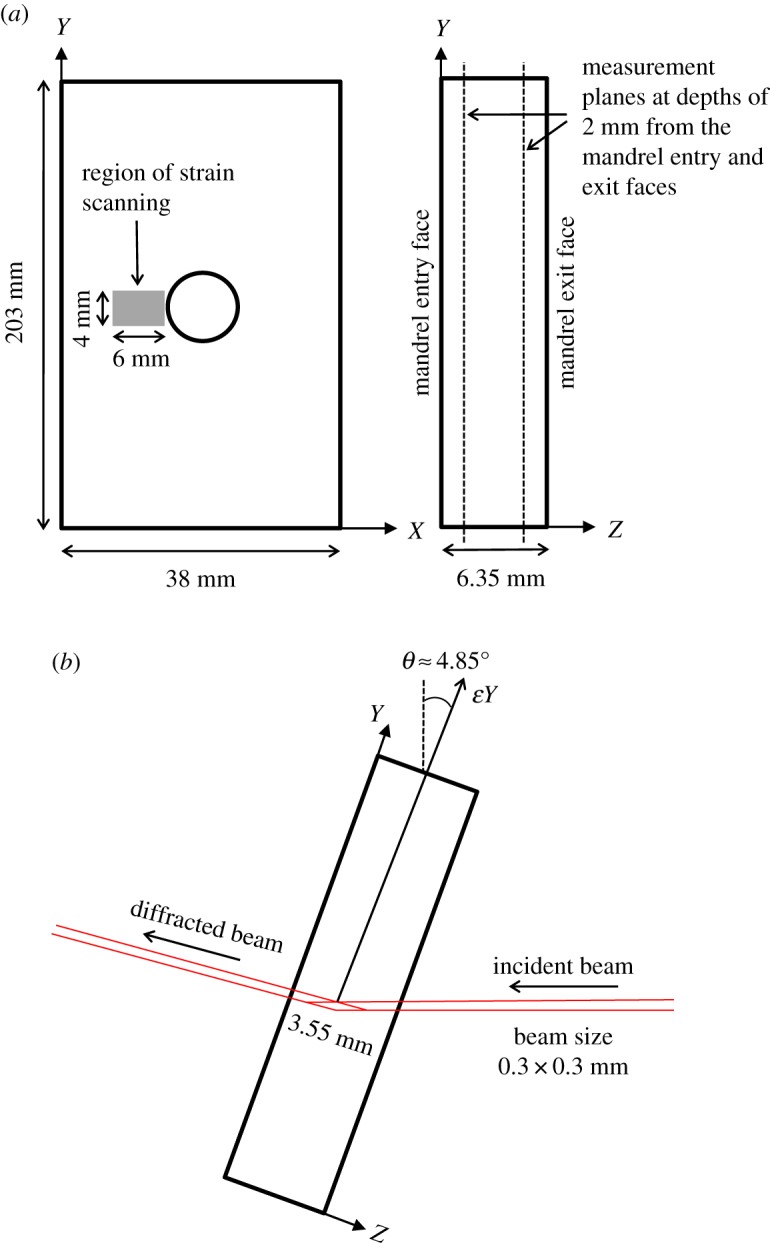
Schematic (*a*) represents the area and measurement planes over which the residual strain scanning was performed in cold-expanded specimens, C8 with a 2.1 mm crack, C9 with a 3.8 mm crack and C12 to which no loads were applied and (*b*) represents the specimen orientation and diffraction geometry for measuring residual strains along *Y* direction (not to scale).

The residual strain scanning was performed on a high-resolution powder diffraction beamline, ID22 at the European Synchrotron Radiation Facility (ESRF). The key objectives were: (i) to determine the initial residual stress distribution developed from cold expansion, (ii) to evaluate the uncertainty in the residual stresses, and (iii) to measure the residual stresses in the specimens, which had been loaded in fatigue, for evidence of any significant residual stress relaxation or redistribution. The residual strains in the longitudinal (*Y*) and transverse (*X*) directions were measured in the region surrounding the fatigue crack at the hole edge in specimens, C8 and C9. A rectangular matrix of 180 measurement points was defined in this region with a uniform spacing of 0.4 mm. The area was scanned twice over two measurement planes, which were defined at a depth of 2 mm from both the mandrel entry and exit faces of the specimen (figure 3*a*). For the purpose of quantitative comparison, the residual strains were also measured in the same region in one of the un-cracked cold-expanded specimens, C12. In order to determine the uncertainty in the initial residual strains, scans along the transverse centre line were performed in specimens C13–C17 at a depth of 2 mm from the mandrel exit face. Similar line scans were also performed in specimens C7, C10 and C11 at a depth of 2 mm from both the mandrel entry and exit faces.

All measurements were performed in a transmission geometry using a monochromatic X-ray beam of 0.3 × 0.3 mm with a photon energy of 60 keV. At this energy, the diffraction from the (311) plane was at 2*θ* ≈ 9.7° approximately. The (311) plane was selected for residual strain measurements because it has been reported as the most suitable plane for representing macroscopic residual strains in an aluminium material [30], and a majority of the investigations [15,31–35], which studied macroscopic residual strains in aluminium materials, have performed diffraction measurements by selecting this plane. Each specimen was mounted on a three-axis translation stage; and, for each measurement, the diffractometer was swept through a fixed 2*θ* angular range from 9.6 to 9.85°, to measure the diffracted intensity peak. To resolve the residual strain components along two orthogonal directions, the specimens were mounted in two different orientations. A schematic of the specimen orientation for measuring residual strain along the *Y* direction is shown in figure 3*b*. The measurement volume, commonly referred to as the gauge volume, is defined by the intersection of the incident and diffracted beams. Low diffraction angles at high X-ray photon energies, as dictated by Bragg's Law, result in an elongated diamond-shaped gauge volume. The dimensions of the gauge volume used in this experiment, resulting from the beam size and the diffraction geometry, are also provided in figure 3*b*.

The strain-free (311) plane spacing, *d*_0_ for aluminium was measured using comb-teeth shaped specimens, which were manufactured using EDM from the same plate as the fatigue specimens. The machining of the comb-teeth shaped structure using EDM relieved any macro-scale residual stresses along the teeth profile, which makes them suitable for the *d*_0_ measurements [36]. The values for *d*_0_ along longitudinal and transverse directions were found to be 1.221679 ± 0.000206 Å and 1.220445 ± 0.000032 Å, respectively. Measured residual strains were converted to stresses using the following form of Hooke's Law assuming plane stress conditions and values of 72 GPa and 0.33 for Young's modulus (*E*) and Poisson's ratio (*v*), respectively:

3.1a $$\begin{array}{rl} \sigma_{x} & =\frac{E}{1-v^{2}}(\varepsilon_{x}+v\varepsilon_{y}) \end{array}$$

3.1b $$\begin{array}{rl} \mathrm{and}\;\;\sigma_{y} & =\frac{E}{1-v^{2}}(\varepsilon_{y}+v\varepsilon_{x}). \end{array}$$

The standard deviations were obtained from a total of 18 measurements along each direction and correspond to an uncertainty of ±169 and ±27 µ-strain in the longitudinal and transverse residual strains, respectively. Stefanescu *et al*. [15], who utilized SXRD in one of their studies, reported the uncertainty of ±74 µ-strain, resulting from variation in *d*_0_, in the residual strain measurements around cold-expanded holes. The vertical and horizontal beam dimensions employed in their work ranged from 0.3 to 1 mm and from 0.6 to 2 mm respectively, giving a lower spatial resolution in comparison with the measurements performed in this work, where the beam size of 0.3 × 0.3 mm was used. However, the strain resolution of ±169 µ-strain for the *Y* direction residual strains was lower in this work compared with ±74 µ-strain in the study by Stefanesu *et al*. [15]. Nonetheless, the strain resolution was high enough to discern any potential residual strain relaxation due to fatigue crack propagation, which was reported to be of the order of 2400 µ-strain close to the hole edge by Stefanescu *et al*. [15]. It is pertinent to mention here that a detailed uncertainty analysis, taking into account the residual stresses due to cold expansion process variability, has not been carried out in any of the previous investigations and is essential to identify any significant redistribution or relaxation of such stresses. Therefore, the propagated uncertainties were evaluated in this work considering the influence of both the variation in *d*_0_ and the cold expansion process as discussed in the later section.

## 4. Results and discussion

A summary of fatigue lives is provided in table 3 for the specimens tested. The average fatigue life of the six cold-expanded specimens is 3.2 times higher than that of the six un-expanded specimens. This improvement is evident despite the fact that the maximum remote stress during fatigue loading for the cold-expanded specimens was 170 MPa compared to 150 MPa for the un-expanded ones. The number of cycles to failure for specimens C10 and C11, to which a single compressive stress cycle of −92.7 and −125 MPa were applied, respectively, are substantially lower than the mean fatigue life of the standard six cold-expanded specimens. The difference is greater than three standard deviations, which clearly indicates that the initial residual stress distribution was significantly relaxed by the applied compressive loads in these specimens.

**Table 3.** Summary of fatigue test results for the un-expanded and the cold-expanded specimens.

un-expanded specimens										
	U1	U2	U3	U4	U5	U6	mean	median	range	s.d.
cycles to failure, *N*_f_ (×10^3^)	86.8	126	99.6	107.6	133.3	89.1	107.1	103.6	46.5	±19.2
cycles to first crack, *N*_I_ (×10^3^)	80.2	92.5	93.5	100.9	106.8	79.6	92.3	93.2	26.6	±10.9
length of first crack observed, *a_i_* (mm)	0.6	0.7	0.9	0.8	0.5	1.1	0.8	0.8	1.2	±0.24
cold-expanded specimens to which no single compressive load cycle was applied										
	C1	C2	C3	C4	C5	C6	mean	median	range	s.d.
cycles to failure, *N*_f_ (×10^3^)	323.4	410.9	288	357.6	342.5	345.0	344.6	343.8	122.9	±40.6
cycles to first crack, *N*_I_ (×10^3^)	114.2	245	89.8	86.3	94.2	103.3	122.1	98.8	155.2	±61
length of first crack observed, *a_i_* (mm)	0.5	0.6	0.4	0.5	0.5	0.5	0.5	0.5	0.2	±0.06
cold-expanded specimens to which single compressive load cycle was applied										
	C10	C11								
cycles to failure, *N*_f_ (×10^3^)	208.2	130.3								
cycles to first crack, *N*_I_ (×10^3^)	104.9	74.2								
length of first crack observed (mm)	0.6	0.5								

### 4.1. Behaviour of fatigue crack propagation

**Figure 4.**
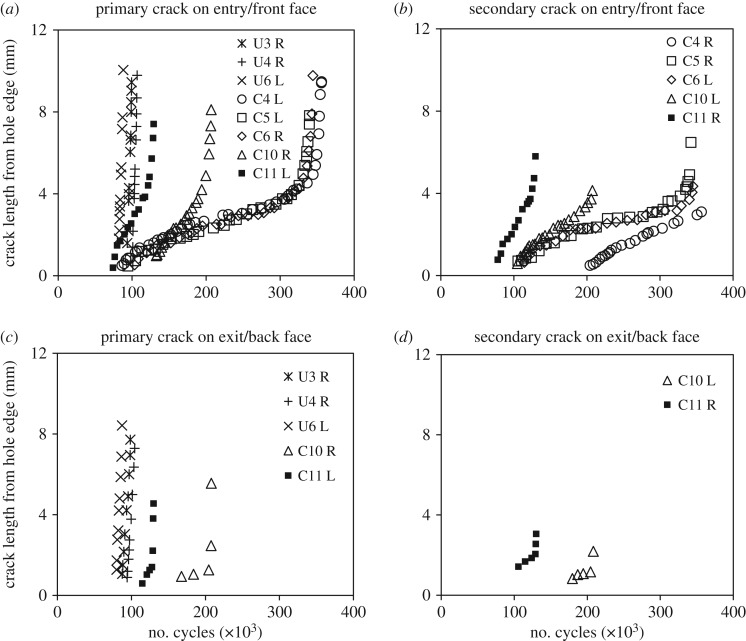
Crack growth plots for (*a,c*) the primary crack, that led to failure, observed on the mandrel entry/front and exit/back face respectively; and (*b,d*) the secondary crack, initiated at the opposite end of the diameter, observed on the mandrel entry/front and exit/back face respectively. Letters, L and R in the plot legends refer to the cracks originating from either left or right side of the hole edge, respectively.

**Figure 5.**
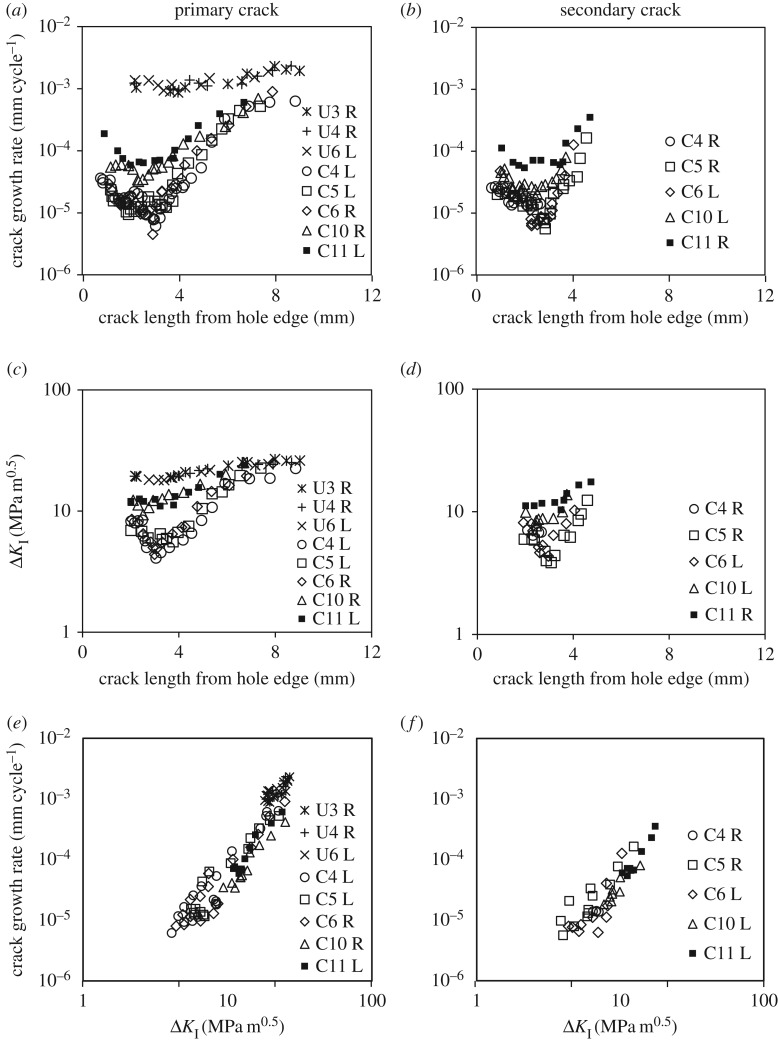
Plots of (*a,b*) crack growth rate obtained by differentiating the growth plots in figure 4*a,b*, respectively; (*c,d*) effective stress intensity factor range determined from TSA data; and (*e,f*) crack growth rate against the effective stress intensity factors. All the plots in this figure are for cracks observed on the mandrel entry/front face of the specimens. Letters, L and R in the plot legends refer to the cracks originating from either left or right side of the hole edge, respectively.

To further investigate and characterize the typical behaviour of both types of holes, the test data for the three cold-expanded specimens (C4, C5 and C6) are plotted along with those for the three un-expanded ones (U3, U4 and U6) in figures 4, 5 and 7. These specimens were selected because their number of cycles to failure was closest to the mean fatigue life of their respective batches. These figures also contain the data for specimens, C10 and C11, to illustrate the effect of a single compressive load on the subsequent fatigue performance of the specimens with cold-expanded holes. The crack growth plots for the above-mentioned specimens are provided in figure 4. For the un-expanded specimens, a single crack initiated from either the left- or right-hand edge of the hole on the transverse axis and appeared almost simultaneously on both faces of the specimen. No crack initiated from the opposite edge of the hole until the specimen failed. In the cold-expanded specimens, a crack first initiated from one end of the transverse diameter of the hole followed by a second crack at the opposite end. The crack that was the longer of the two, which eventually led to specimen failure, is referred to as the primary crack and the other crack, initiated at the opposite end, is referred to as the secondary crack. Both the cracks appeared only on the mandrel entry faces of these specimens, which was expected because the compressive residual stresses are lower in magnitude on the entry face compared with the exit face. The experimental measurements had revealed that the magnitude of residual hoop stress, close to the hole edge, on the mandrel entry face could be lower by 7%–50% of the residual hoop stress value on the exit face [37–42]. The primary reason for this through-thickness variation in the residual stresses is believed to be the difference in the constraint conditions between the two faces during cold expansion. However, there are other factors which are likely to influence this as well, such as the thickness-to-diameter ratio, cold expansion level and the type of process used for cold expansion. In specimens C10 and C11, cracks propagated from both ends of the transverse diameter of the hole and appeared first on the mandrel entry face and later on the exit face. It can be seen from the crack growth plots that, for almost all the specimens, the first crack was observed at about 100 k cycles. This indicates that cold expansion improves the fatigue performance by retarding the crack growth rather than delaying the crack initiation, which is in agreement with the findings reported by Chandawanich & Sharpe [1]. The information about the number of cycles at which the first crack was observed (*N_i_*) and its length (*a_i_*) is provided for all the specimens tested in table 3.

**Figure 6.**
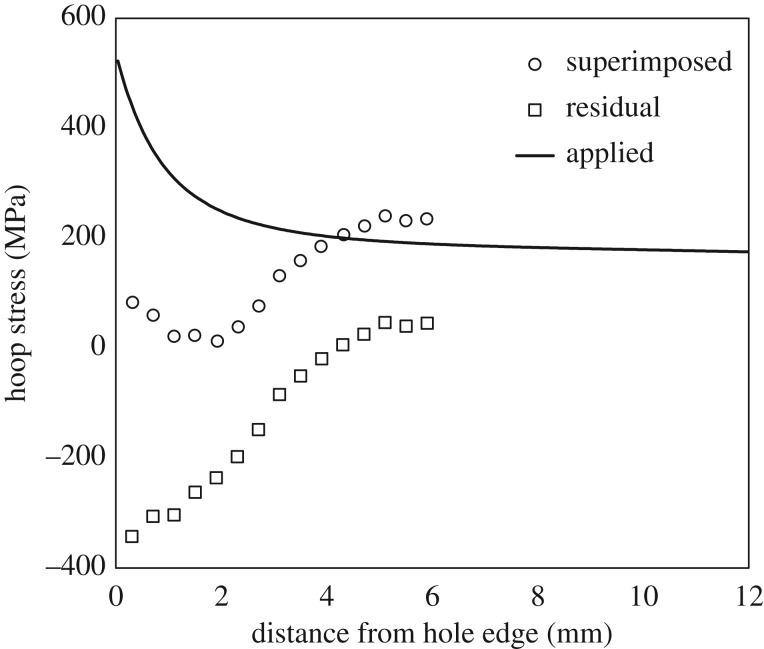
Plot of superimposed hoop stress profile along the transverse centre line of the specimen which was determined by addition of the tensile hoop stress profile resulting from the applied remote stress of 170 MPa and the compressive residual hoop stress profile developed by cold expansion.

The crack growth profiles for the mandrel entry/front face in figure 4*a,b* were differentiated by fitting a least-squares regression line to every five consecutive data points to obtain the growth rate plots shown in figure 5*a,b*. In cold-expanded specimens C4–C6, the cracks started off with a relatively higher crack growth rate of 0.03 µm cycle^−1^, which decreased to a minimum value of 0.007 µm cycle^−1^ before increasing again. This characteristic trend is consistent in all three specimens for the cracks emanating from both ends of the transverse diameter through the hole. The average distance from the hole edge of the turning points in the crack growth rate plots was found to be 2.7 mm for the primary cracks and 2.2 mm for the secondary cracks in the three specimens. This characteristic trend is believed to result from a combined effect of decreasing applied stresses due to the reduced influence of the stress concentration and the presence of a compressive residual stress distribution, which reduces to zero at 1 mm beyond the location of the turning point. To illustrate this, a superimposed hoop stress profile along the transverse centre line of the specimen is shown in figure 6, which was determined by superimposing the tensile hoop stress profile resulting from the remote stress of 170 MPa on the compressive residual stress profile determined from SXRD. As expected, the crack growth rate profiles in figure 5*a,b* are in reasonable correlation with the superimposed hoop stress profile in figure 6. A similar trend could also be observed in specimens C10 and C11; but the turning points are progressively less pronounced, indicating increasing levels of residual stress relaxation or redistribution in the two specimens. By contrast, the crack growth rates are consistently very high, of the order of 1.4 µm cycle^−1^, for the three un-expanded specimens in the absence of any compressive residual stresses.

Several researchers [1–7,18] attempted to determine Δ*K*_eff_ for cracks emanating from cold-expanded holes. Almost all of them [1–7] used a theoretical approach, in which Δ*K* solutions for the residual stress field and the applied stresses are superimposed to evaluate Δ*K*_eff_. However, a few researchers [1–3] also compared their theoretical predictions of Δ*K*_eff_ with experimental ones and found reasonable agreement. The experimental values of Δ*K*_eff_ were derived from the recorded crack growth rates using existing d*a*/d*N *− Δ*K* databases. In this work, Δ*K*_eff_ values were obtained directly from the thermoelastic data using the methodology of Tomlinson *et al*. [21] briefly described in an earlier section. This approach requires thermoelastic data collection from the singularity-dominated region surrounding the crack tip, which makes it difficult to apply for small cracks close to the hole edge. For this reason, Δ*K*_eff_ was evaluated when the cracks were at least 2 mm in length. The trends in the Δ*K*_eff_ plots for the primary and the secondary cracks shown in figure 5*c,d* appear to be consistent with those of the crack growth rate plots in figure 5*a,b*, respectively. The crack growth rates are plotted against Δ*K*_eff_ in figure 5*e,f* and it can be seen the data for all of the specimens form a single curve.

### 4.2. Plastic zones associated with fatigue crack tip

**Figure 7.**
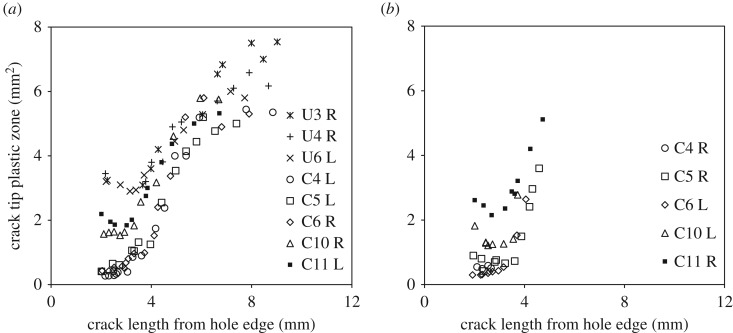
Plots of crack tip plastic zone area for (*a*) primary and (*b*) secondary cracks observed on the mandrel entry/front face of the specimens, as in figure 4*a,b*, respectively.

**Figure 8.**
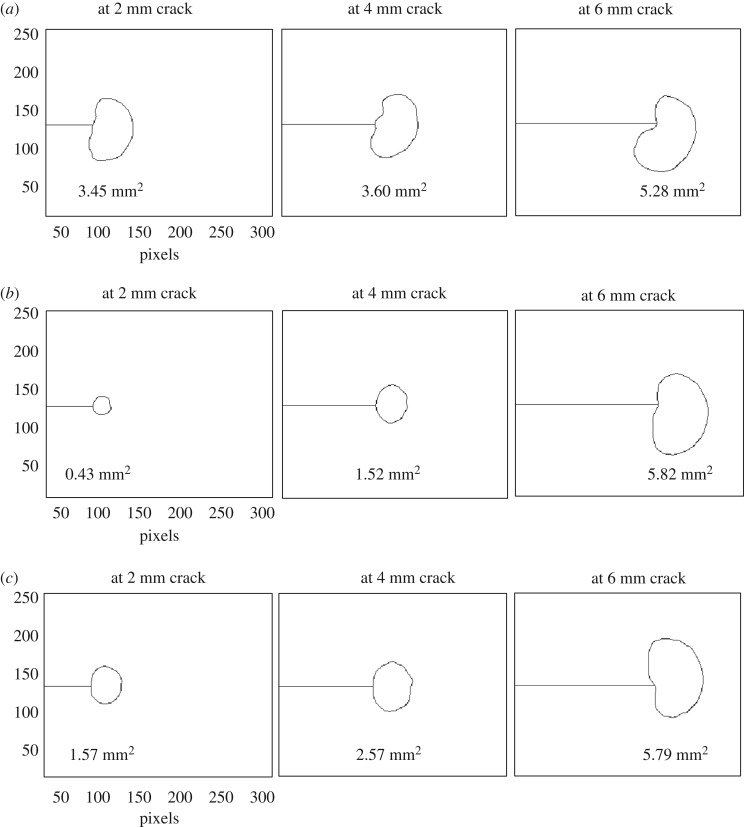
Plastic zones associated with the crack tip at three different lengths of the crack that led to failure for (*a*) un-expanded specimen, U3, (*b*) cold-expanded specimen, C6 to which no initial compressive stress cycle was applied and (*c*) cold-expanded specimen, C10 to which single compressive stress cycle of −92.7 MPa was applied prior to fatigue loading. The spatial dimensions of the maps represent the infrared camera sensor window of 256 × 320 pixels (1 pixel ≈ 0.03 mm).

The map of phase difference between the measured thermoelastic signal and the loading signal from the servo-hydraulic test machine can be used to identify regions on the specimen's surface where adiabatic conditions have been lost due to heat generation associated with plastic deformation. This phase difference information has been used successfully in the past by Patki & Patterson [43] and, more recently, by Yang *et al*. [44] to determine the extent of the cyclic plastic zone associated with the crack tip. It was also utilized in this work to measure the shape and size of the crack tip plastic zones for cracks emanating from both the un-expanded and the cold-expanded holes. The plots representing the variation in plastic zone size with increasing crack length are given in figure 7. For cold-expanded specimens C4–C6, to which no initial compressive load cycle was applied, the plastic zones are consistently much smaller in size until the crack length reaches 4 mm, beyond which, the size of the plastic zone increases rapidly. This reduction of the plastic zone size is due to the presence of compressive residual stresses, which disappear at 4.2 mm from the hole edge (figure 6). To illustrate evolution of the crack tip plastic zone, its shape at three different crack lengths is shown in figure 8 for three specimens: U3, C6 and C10.

**Figure 9.**
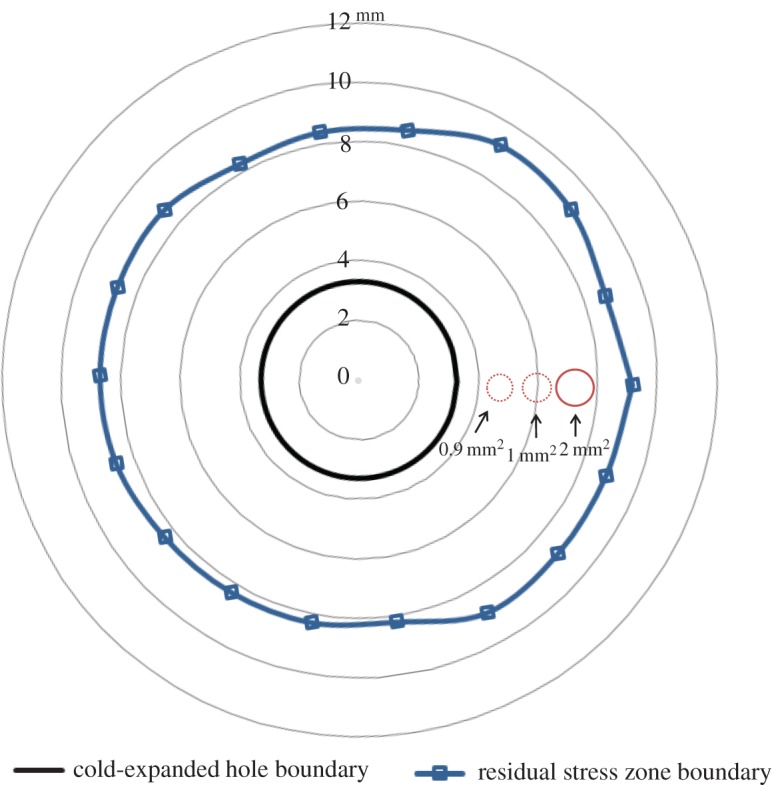
Schematic diagram showing a comparison between the size of residual stress zone, developed on the mandrel entry face by cold expansion, and the plastic zones associated with the crack tip for 2, 3 and 4 mm long cracks.

In a recent article by the current authors [45], the size and shape of the residual stress zone developed from split sleeve cold expansion was measured using the digital image correlation technique and its area, on the mandrel entry face, found to be approximately 220 mm^2^. The plastic zone associated with the crack tip for 4 mm crack was measured to be less than 2 mm^2^, while area of the plastic wake was of the order of 3 mm^2^, based on the size of the crack tip plastic zone at shorter crack lengths. The schematic in figure 9 shows a comparison of the two zones. The diameter of the crack tip plastic zone is 28% of the annular thickness of the residual stress zone and its size is about 1% of the overall area of the residual stress zone, which rises to 2.5% when the crack wake plastic zone is included. This demonstrates that, due to the large extent of the residual stress zone, the residual stresses induced by cold-working, which surround the crack geometrically, act as remote stresses; and the localized plastic zone associated with the crack tip is not sufficiently significant in size to cause the displacements required for relaxation of these residual stresses. To further investigate this hypothesis, which is based on results from TSA, residual stresses were measured in both the un-cracked and the cracked cold-expanded specimens using SXRD.

### 4.3. Effect of fatigue crack propagation on the residual stresses

**Figure 10.**
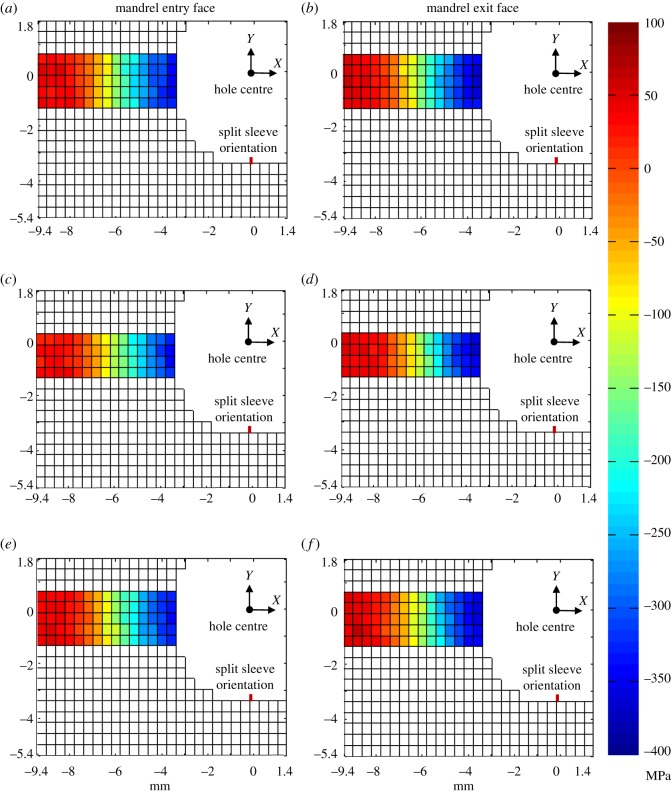
Maps of *Y* component of residual stresses close to the mandrel entry (left) and exit (right) face for (*a,b*) cold-expanded specimen, C12 to which no loads were applied; (*c,d*) cold-expanded specimen, C8 with a 2.1 mm crack; and (*e,f*) cold-expanded specimen, C9 with a 3.8 mm crack.

**Figure 11.**
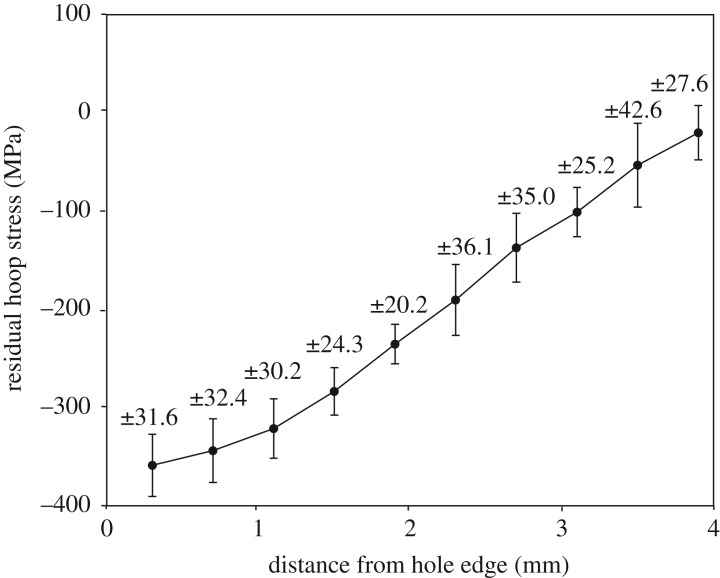
Average residual stress profile, close to the mandrel exit face, for cold-expanded specimens, C12–C17 to which no loads were applied.

Figure 10 shows maps of the residual stresses measured at a depth of 2 mm from both the faces in specimens C8, C9 and C12. Only the longitudinal (*Y*) component of the residual stresses is presented as they act perpendicular to the crack, thereby playing a dominant role in the mechanism of fatigue crack propagation. Prior to SXRD measurements, specimens C8 and C9 were loaded in fatigue to grow cracks of length 2.1 and 3.8 mm, respectively, which were observed on the mandrel entry face of these specimens. There does not seem to be any significant residual stress relaxation in specimens C8 and C9 as their stress fields appear to be very similar to those of the un-cracked specimen, C12. To perform a detailed uncertainty analysis, taking into account the variation in the strain-free lattice spacing and the cold-expansion process, residual stresses were measured in six un-cracked specimens, C12–C17, along the transverse centre line at a depth of 2 mm from mandrel exit face. The average residual stress distribution for the six specimens is shown in figure 11. The values for the propagated uncertainty were calculated based on the principles defined in the guide to measurement uncertainty [46] and are shown as 95% confidence limits for the mean value, or two standard deviations.

**Figure 12.**
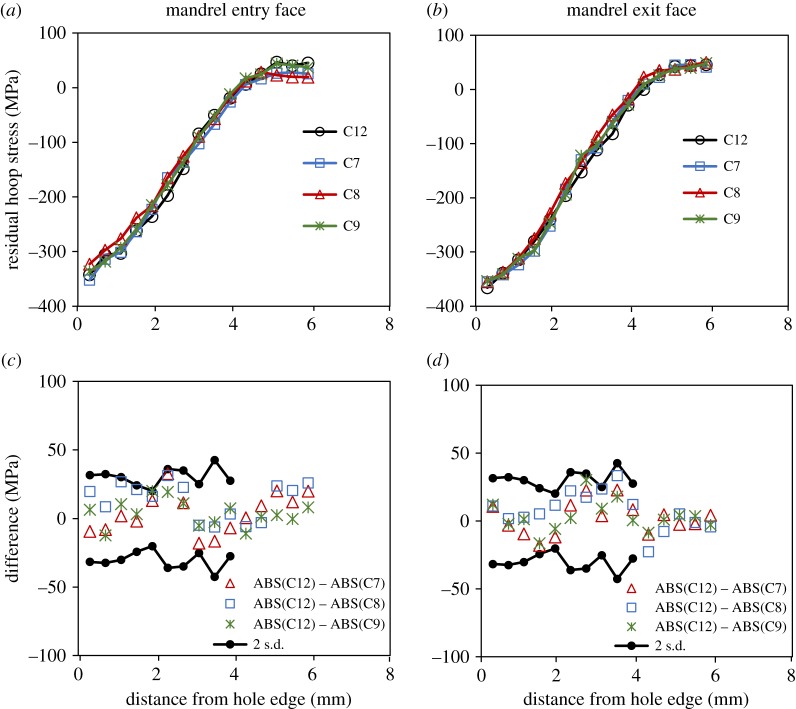
Plots of (*a,b*) residual stress profiles along the transverse centre line of the specimen and (*c,d*) difference in their magnitudes close to the mandrel entry and exit faces for cold-expanded specimens, C7 to which 50 k cycles of fatigue loading was applied with no cracks observed during loading, C8 with a 2.1 mm crack, C9 with a 3.8 mm crack and C12 to which no loads were applied.

For a more quantitative comparison of the measured residual stresses in specimens C8, C9 and C12, their distributions are plotted in figure 12*a,b* along the crack line, which also coincides with the transverse centre line of the specimen. The plot also includes the distribution for specimen, C7 to which 50 k cycles of fatigue loading was applied, with the purpose of determining whether fatigue loading on its own brings about any redistribution of residual stresses prior to initiation of a primary fatigue crack, as reported by Özdemir & Edwards [13]. The residual stress profiles for specimens C7–C9 were subtracted from those of the un-cracked specimen, C12 and the differences are plotted in figure 12*c,d*. All the values are within the propagated uncertainty bounds, which were obtained from the uncertainty analysis of the measurements made in the six un-cracked specimens, C12–C17. This clearly shows that there is no significant relaxation of residual stresses resulting either from fatigue loading or due to propagation of a fatigue crack. This reinforces the conclusions drawn from the TSA results that there is negligible influence of crack tip plastic zone on the surrounding residual stresses. In contrast to the findings of Stefanescu *et al.* [15], the plots in figure 12*a,b* do not show any pronounced relaxation, even at the hole edge. In their work, cracks were grown from an EDM notch, whereas in this work, the fatigue cracks were initiated naturally as a result of fatigue loading. This supports the argument that installing a notch involves material removal and associated plastic work together with the creation of a geometric discontinuity resulting in a relaxation of the residual stresses around the edge of the hole.

### 4.4. Effect of compressive loads on the residual stresses

**Figure 13.**
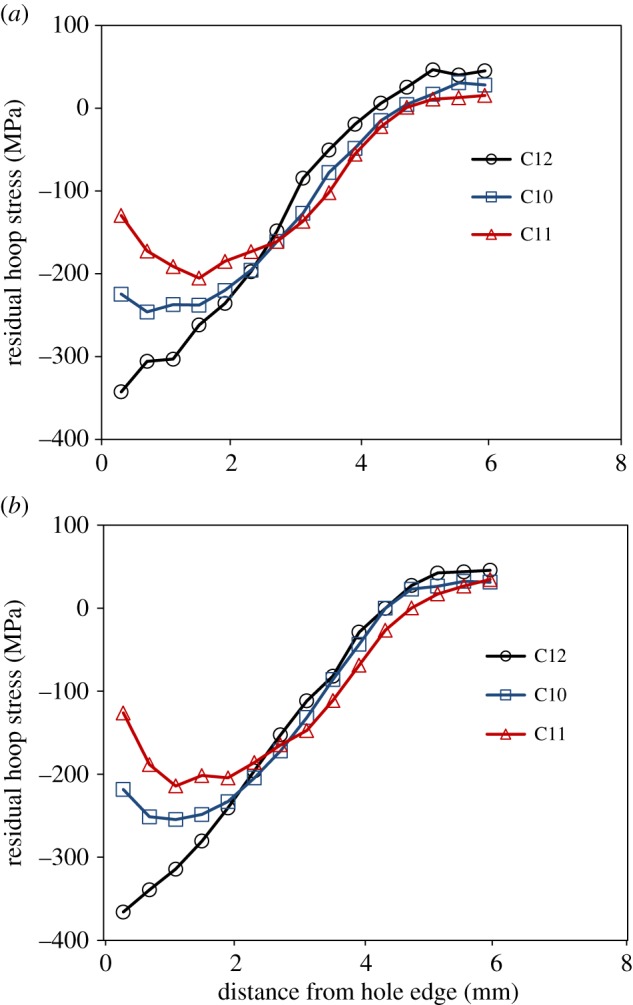
Plots of residual stress profiles close to (*a*) the mandrel entry face and (*b*) the mandrel exit face for cold-expanded specimens, C10 to which single compressive stress cycle of −92.7 MPa was applied, C11 to which single compressive stress cycle of −125 MPa was applied and C12 to which no loads were applied.

For specimens, C10 and C11, the maximum hoop stress at the hole edge, *σ*_h,max_ due to the single applied compressive load was calculated to be −627 and −730 MPa, respectively, which is substantially higher than the ultimate tensile strength of the material i.e. 505 MPa. The 2024-T351 aluminium plate material used in this research is known to have slightly lower yield and ultimate strengths in compression in comparison to tension [47]. This implies that there will be large-scale plastic deformation causing a redistribution of the initial residual stresses. Figure 13 shows the adversely affected residual stress profiles for specimens C10 and C11, which suggests an explanation for the higher crack growth rates; and consequently, higher Δ*K*_eff_ values and larger crack tip plastic zones in these specimens, as shown in figures 5 and 7. The stress relaxation at the hole edge was found to be 34% and 62% close to the mandrel entry face and 40 and 66% close to the exit face for specimens C10 and C11, respectively. It was expected that there would be a slightly higher relaxation on the exit face due to the higher magnitude of the compressive residual stresses on the exit face in comparison to the entry face.

### 4.5. Effect of residual stresses on fatigue crack initiation

**Figure 14.**
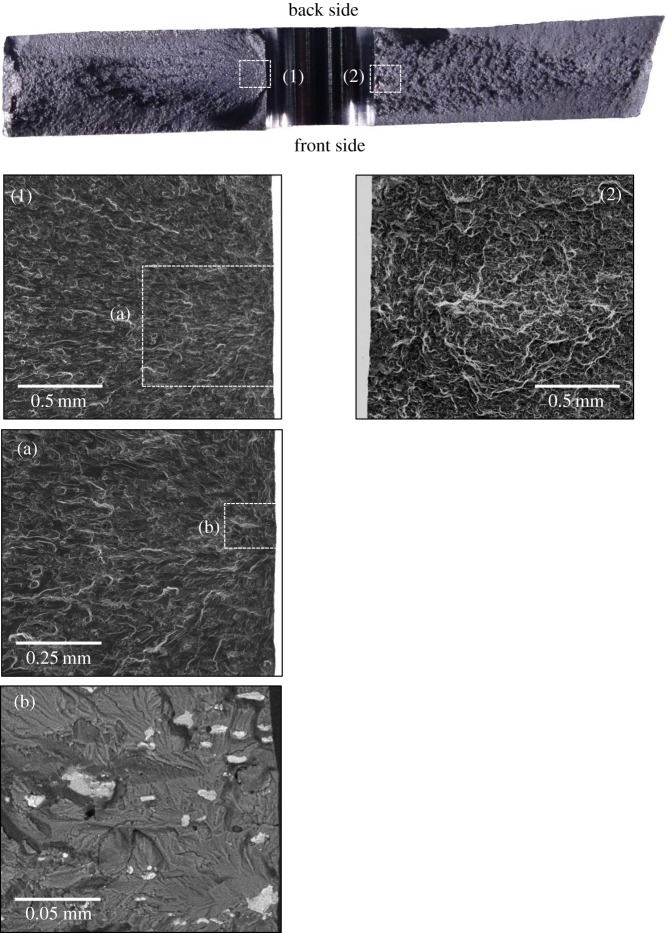
Fracture surface morphology of the un-expanded specimen, U6. The optical micrograph at the top shows the whole fracture surface. SEM images on the left show the origin of fatigue crack initiating from the left edge of the hole. SEM image on the right highlights the typical morphology which results from fast fracture.

**Figure 15.**
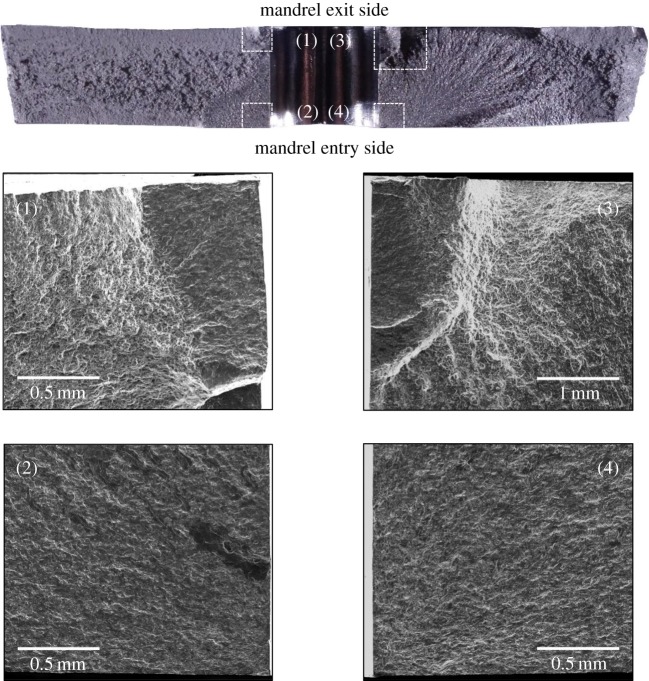
Fracture surface morphology of the cold-expanded specimen, C6 with unmodified residual stress distribution. The optical micrograph at the top shows the whole fracture surface. SEM images 1 and 3 show the origin of secondary fatigue cracks initiating from left and right corners on the mandrel exit side, respectively. SEM images 2 and 4 show the origin of primary fatigue cracks initiating from left and right corners on the mandrel entry side, respectively.

For qualitative analysis of the effect of residual stresses on fatigue crack initiation, the fatigue features on the fracture surface were observed in three specimens: U6, C6 and C10, using both an optical and scanning electron microscope (SEM). The optical images of the whole fracture surface were obtained using a stereo microscope (SZ61; Olympus, Japan), whereas the images of crack initiation sites on the fracture surface were recorded using an SEM (JSM-7610F; JEOL Ltd, Japan). Figure 14 shows the fractographs of specimen, U6. The data recorded using TSA and a pair of digital cameras, during the fatigue tests, indicated the appearance of a fatigue crack on both the specimen faces only on the left side of the hole (figure 4) and the specimen fractured due to overload of the right-side ligament. The fatigue crack growth (FCG) region on the left side can be easily distinguished from a fast fracture region on the right by its surface roughness. The right-side surface is much rougher indicating significant plastic deformation due to overload. Focusing on the FCG region, it can be observed from SEM image 1 in figure 14 that the features converge to a single location at the hole edge, on the mid-plane of the specimen, highlighting the point of initiation of the fatigue crack. The shape of the features also indicates that the crack grew with a semi-circular front. A higher magnification back-scattered electron image (b) shows the presence of intermetallic particles at the hole edge which probably is the reason for fatigue crack initiation from this site as there is no evidence of machining marks along the hole edge. The fractographs of the cold-expanded specimen, C6 are shown in figure 15. The primary fatigue cracks, on both sides of the hole, initiated from the corner on the mandrel entry face (see images 2 & 4) and propagated with a quarter-elliptical crack front. The presence of secondary fatigue cracks can also be seen in images 1 & 3 (in figure 15) at the exit face corners, which are at different depths to the primary cracks. The fibrous features ahead of these secondary cracks represent rupture which occurred during specimen fracture.

**Figure 16.**
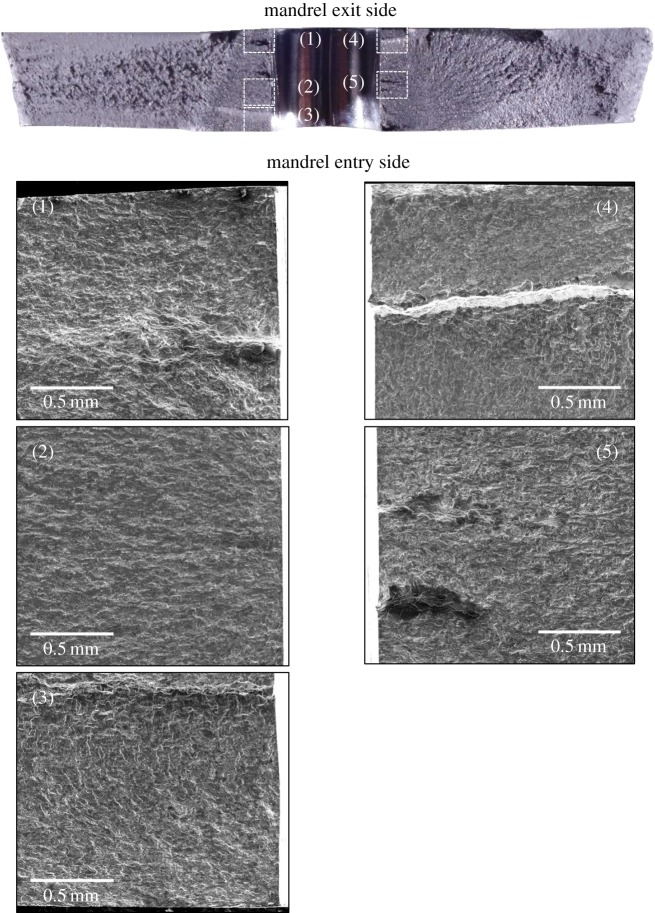
Fracture surface morphology of the cold-expanded specimen, C10 with modified residual stress distribution after a single compressive stress cycle of −92.7 MPa was applied prior to fatigue loading. The optical micrograph at the top shows the whole fracture surface. SEM images on the left and right highlight multiple crack initiation sites along the left and right edges of the hole, respectively.

With reference to open cold-expanded holes under uniaxial fatigue loading, many fractographic investigations [3,48–50] have reported the initiation of a fatigue crack from the corner of the mandrel entry face and the primary reason provided is the lower magnitude of the compressive residual stresses on the mandrel entry face in comparison with the exit face. These investigations involved specimens made of different materials and having different thicknesses, which suggest that the initiation of fatigue cracks from cold-expanded holes is not influenced by the microstructure or the specimen thickness, but is solely governed by the through-thickness distribution of the residual stresses. In a very recent article by Wang *et al*. [50], the fracture surface of a cold-expanded specimen was presented and shows a localized fast fracture zone within the FCG region. The extent of this localized zone was from 0.5 to 2.5 mm from the hole edge and was reported to be bypassed by the fatigue crack due to the presence of tri-axial compressive stresses in this zone. Hence, the reduction in the FCG rate was attributed to this localized fast fracture zone. No evidence of any such localized zone was found from the fractographic analysis performed in this work, but the retardation in crack growth is still evident from figure 5*a,b*. The proposed explanation for crack growth retardation, therefore, is the combined effect of applied stresses that decrease away from hole edge, due to the reduced effect of the stress concentration, and the presence of compressive residual stresses, which the superimposed residual hoop stress profile in figure 6 confirms. To analyse the effect of residual stress relaxation on fatigue crack initiation, the fractographs of specimen, C10 are shown in figure 16. The SEM images of the regions highlighted in white on the optical fractograph show the crack initiation sites. It can be seen that cracks initiated from multiple sites along the hole edge and merged at a later stage to form a single fatigue crack. The resulting crack front appears to be much straighter in comparison to the one developed in the cold-expanded specimen with the unmodified residual stress distribution (specimen C6 in figure 15). This implies that the though-thickness variation in the modified residual stresses is significantly lower, which the SXRD measurements confirm. The difference between the residual hoop stresses close to the entry and exit faces at the hole edge was found to be 30 MPa for the initial, and 4 MPa for the modified, residual stress distribution.

Cold expansion is usually performed in aerospace materials such as aluminium, titanium and steel alloys. Despite having significantly different mechanical properties, they all exhibit similar strain hardening; and hence, the three-dimensional residual stress field developed as a result of cold expansion will have a similar form in these materials. The major difference will be in the magnitude and extent of the residual stress field, which is dictated by the mechanical properties of the material. The findings presented in this article provide some meaningful insights into the mechanism of fatigue crack propagation through a highly compressive residual stress field and the interaction of the crack tip plastic zone of a growing crack with the surrounding residual stresses. They also clearly demonstrate that the behaviour of fatigue crack propagation is governed primarily by the three-dimensional distribution of residual stresses; which, as mentioned above, is similar for the commonly used aerospace materials. This implies that the conclusions drawn from TSA and SXRD results should not be restricted to the particular grade of aluminium alloy material investigated in this work. It was also established that the initial residual stress distribution developed from cold expansion undergoes redistribution when the applied loads are large enough to cause yielding at the edge of cold-expanded holes. A simple approach of linear superposition of hoop stress at the hole edge resulting from the applied load and the compressive residual stress can be used as an initial estimate to determine whether a given applied load is expected to cause yielding at the hole edge. In this work, uniaxial compressive loads have been used to demonstrate residual stress redistribution but this simple approach for determining the potential for a given load to cause residual stress redistribution can be extended to other loading scenarios. Nonetheless, the information provided in this article about the potential for and causes of any redistribution of beneficial compressive residual stresses developed from cold expansion is important in improving the theoretical models for fatigue life assessment of cold-expanded holes. It would also be useful for the engineers in the aerospace industry to realize the full potential of the cold-expansion process and to utilize it more effectively in the manufacturing of airframes leading to improved fatigue endurance under different loading conditions.

## 5. Conclusion

The study presented in this paper has used thermoelastic stress analysis (TSA) and synchrotron X-ray diffraction (SXRD) techniques to analyse the behaviour of fatigue cracks emanating from cold-expanded holes and their influence on the surrounding residual stresses, which extended to about 6 mm from the edge of the hole. A characteristic trend was observed for cracks initiating from cold-expanded holes: namely that, the crack growth rate decreased to a minimum at approximately 3 mm from the hole edge beyond which it increased as the crack grew out of the influence of the residual compressive stresses associated with cold expansion. The TSA results showed that the plastic zones associated with the crack tip were reduced in size significantly by the presence of these compressive residual stresses. For a 4 mm crack on the mandrel entry face, the diameter of the crack tip plastic zone was calculated to be 28% of the annular thickness of the residual stress zone, and, moreover, its size was found to be about 1% of the overall area of the residual stress zone resulting from cold expansion. This implies that the crack tip plastic zone is not sufficiently significant in size to cause the deformation required for relaxation or redistribution of these beneficial residual stresses. To validate this hypothesis based on TSA results, residual stresses were measured around both un-cracked and cracked cold-expanded holes using SXRD. No sign of any significant residual stress relaxation was found in the cold-expanded specimens in which a fatigue crack had initiated. Therefore, in contrast to the previously published findings [13–15], the results from TSA and SXRD clearly demonstrate that the residual compressive stresses do not relax or redistribute as a result of fatigue loading or due to the propagation of fatigue cracks, as long as the applied loads are not high enough to cause large-scale plastic deformation at the hole edge.

An investigation was also conducted of the influence on the initial residual stress distribution of applied loads that were large enough to cause local yielding at the edge of the hole. As a consequence of the compressive nature of the residual stresses, only a relatively small applied single compressive load was required to cause yielding. The SXRD measurements revealed substantial relaxation of the initial residual stress distribution due to the application of a compressive load. These results clearly highlight the loading conditions under which the beneficial compressive residual stresses are expected to relax. This information is likely to be significant in improving the fatigue life prediction models for cold-expanded holes and for other scenarios where cold-working is used to induce beneficial residual stresses.

## Data accessibility

The experimental data and the Matlab code for determining the shape and size of the crack tip plastic zone can be found at the Dryad Digital Repository [51]: https://doi.org/10.5061/dryad.51256.
